# The EphB4 Receptor Tyrosine Kinase Promotes Lung Cancer Growth: A Potential Novel Therapeutic Target

**DOI:** 10.1371/journal.pone.0067668

**Published:** 2013-07-02

**Authors:** Benjamin D. Ferguson, Ren Liu, Cleo E. Rolle, Yi-Hung Carol Tan, Valery Krasnoperov, Rajani Kanteti, Maria S. Tretiakova, Gustavo M. Cervantes, Rifat Hasina, Robyn D. Hseu, A. John Iafrate, Theodore Karrison, Mark K. Ferguson, Aliya N. Husain, Leonardo Faoro, Everett E. Vokes, Parkash S. Gill, Ravi Salgia

**Affiliations:** 1 Pritzker School of Medicine, University of Chicago, Chicago, Illinois, United States of America; 2 Department of Medicine, Section of Hematology/Oncology, University of Chicago, Chicago, Illinois, United States of America; 3 Department of Pathology, University of Chicago, Chicago, Illinois, United States of America; 4 Department of Surgery, University of Chicago, Chicago, Illinois, United States of America; 5 Department of Health Studies, University of Chicago, Chicago, Illinois, United States of America; 6 Comprehensive Cancer Center, University of Chicago, Chicago, Illinois, United States of America; 7 Department of Medicine, Division of Medical Oncology, University of Southern California, Los Angeles, California, United States of America; 8 Vasgene Therapeutics, Inc., Los Angeles, California, United States of America; 9 Department of Pathology, Massachusetts General Hospital, Boston, Massachusetts, United States of America; Dartmouth, United States of America

## Abstract

Despite progress in locoregional and systemic therapies, patient survival from lung cancer remains a challenge. Receptor tyrosine kinases are frequently implicated in lung cancer pathogenesis, and some tyrosine kinase inhibition strategies have been effective clinically. The EphB4 receptor tyrosine kinase has recently emerged as a potential target in several other cancers. We sought to systematically study the role of EphB4 in lung cancer. Here, we demonstrate that EphB4 is overexpressed 3-fold in lung tumors compared to paired normal tissues and frequently exhibits gene copy number increases in lung cancer. We also show that overexpression of EphB4 promotes cellular proliferation, colony formation, and motility, while EphB4 inhibition reduces cellular viability *in vitro*, halts the growth of established tumors in mouse xenograft models when used as a single-target strategy, and causes near-complete regression of established tumors when used in combination with paclitaxel. Taken together, these data suggest an important role for EphB4 as a potential novel therapeutic target in lung cancer. Clinical trials investigating the efficacy of anti-EphB4 therapies as well as combination therapy involving EphB4 inhibition may be warranted.

## Introduction

Although numerous drug targets have been studied extensively, the overall survival of lung cancer has improved only minimally over the past four decades [1]. One frequent characteristic of lung cancer is an aberration in which one or more receptor tyrosine kinases (RTKs), such as MET, EGFR, and ALK, are commonly overexpressed, amplified, or mutated [2]–[4]. The Eph family is the largest family of RTKs, comprising fourteen mammalian receptors that interact with eight mammalian ligands, or ephrins. Eph receptors and ephrin ligands are organized functionally and structurally into A- and B-classes [5]. Eph receptors are relatively similar across classes; however, the membrane-bound ephrin-B ligands are unique in that they possess extracellular domains that activate receptors as well as intracellular domains that are thought to signal downstream within an adjacent cell [6]. There is some promiscuity among receptor and ligand interactions; one of the most specific receptor-ligand interactions, however, is between EphB4 and ephrin-B2 [7].

Classically, Eph receptors have been major players in developmental biology given their roles in axon guidance, vasculogenesis, and neural development [8]. However, several Eph receptor family members have also been shown to play a role in cancer. We have recently shown that EphA2 is overexpressed in lung cancer and harbors a gain-of-function point mutation in some squamous cell carcinomas, and EphA2 inhibition in addition to rapamycin exposure in lung cancer cells reduced their proliferation *in vitro* [9]. EphB4 has also been reported to play an oncogenic role in cancers of the head and neck [10], [11], prostate [12], [13], other genitourinary organs [14]–[19], breast [20], mesothelium [21], esophagus [22], skin [23], and large bowel [24], [25]. EphB4 mutations have also recently been reported in lung cancer [26], [27], although their significance is currently unknown. However, it has not been systematically studied in lung cancer and its potential role in this disease therefore remains an important question.

Here, we show that EphB4 is an important therapeutic target in lung cancer, as it is overexpressed and often demonstrates increased gene copy numbers. Moreover, knockdown or inhibition of EphB4 attenuates the growth of cancer cells *in vitro* and *in vivo*, whereas introduction of wild-type EphB4 provides a gain of function in tumor cells. Taken together, these data identify EphB4 as a potentially important driver in the pathogenesis of lung cancer.

## Materials and Methods

### Ethics Statement

Tumor tissues were documented along with patient characteristics when available with written informed consent and in accordance with University of Chicago Institutional Review Board-approved protocol. All animal studies were approved by the Institutional Animal Care and Use Committee at the University of Southern California and performed in accordance with Animal Welfare Act regulations.

### Reagents and Antibodies

All primers were designed using Primer3Plus [28] and purchased from Integrated DNA Technologies (Coralville IA). Anti-EphB4 antibodies (#265 for IB and #131 for IHC), anti-ephrin-B2 antibody (#2B5), and human serum albumin-conjugated soluble EphB4 (sEphB4-HSA), were generously provided by the Gill Laboratory, University of Southern California. Ephrin-B2/Fc chimeric protein was purchased from R&D (Minneapolis MN). Anti-ß-actin antibody was purchased from Sigma (St. Louis MO). AZ12489875-002 was a generous gift from AstraZeneca. SN-38 was purchased from Tocris Bioscience (Bristol UK).

### Cell Culture

A549, H358, H522, H661, H1703, H1993, H2170, SW1573, BEAS-2B, H69, H82, H184, H249, H345, H446, H526, H2171, and PC3 cell lines were purchased from American Type Culture Collection (ATCC, Manassas VA. Cell lines were authenticated by ATCC and are routinely tested for the presence of mycoplasma. All cell lines were maintained at 37°C and 5% CO2 in RPMI medium supplemented with 10% fetal bovine serum (FBS), 1% penicillin/streptomycin, 2% sodium bicarbonate, 1% sodium pyruvate, 1% HEPES buffer, and 1% L-glutamine.

### Tissue Procurement

Human lung cancer patient archival tissues were obtained from the University of Chicago Human Tissue Resource Center.

### Immunohistochemistry

Paraffin lung cancer tissue sections were deparaffinized in xylene, rehydrated through graded ethanol solutions to distilled water, and washed in Tris-buffered saline (TBS). Antigen retrieval was carried out by heating sections in citrate buffer (pH 6) for 15 minutes in a microwave. Endogenous peroxidase activity was quenched by incubation in 3% H_2_O_2_ in methanol for 5 minutes. Non-specific binding sites were blocked using Protein Block (Dako, Carpinteria CA) for 20 minutes. Sections were incubated for 1 hour at room temperature with anti-EphB4 primary antibody (anti-mouse; #131) at a concentration of 40 µg/mL, then incubated for 30 minutes with goat anti-mouse IgG conjugated to a HRP-labeled polymer (Bio SB, Santa Barbara CA). Slides were then developed for 5 minutes with 3–3′-diaminobenzidine chromogen, counterstained with hematoxylin, and coverslipped. Negative controls were performed by substituting primary antibody with non-immune mouse immunoglobulins. Colon cancer and brain tissue sections served as positive controls for EphB4 staining [25], [29].

Tissue expression for each sample was quantified by manual scoring on a 0/1+/2+/3+ scale, as well as by an Automated Cellular Imaging System (ACIS; Clarient, Aliso Viejo CA), which quantitates staining intensity based on the ratio of brown to blue pixels per unit area. ACIS software calculates the average intensity for each region analyzed and computes integrated optical density (IOD), which is directly proportional to the concentration of antibody-bound EphB4 molecules according to the Beer-Lambert Law [30]. Therefore, IOD is a proxy for antigen content, and it was normalized to the entire measured area by calculating IOD/10 µm^2^. Overall, manual scoring and ACIS analysis were used as parallel expression quantification methods and were found to have a correlation of r^2^ = 0.75 (Spearman correlation, p<0.0001; Figure S1). As expression trends were similar with both methods, primarily ACIS data are reported here. Expression patterns using the same anti-EphB4 antibody were similar in fresh frozen tissue specimens (Figure S2).

### Immunoblotting

Whole-cell lysates were collected using M-PER mammalian protein extraction reagent (Pierce, Rockford IL) supplemented with 1.8× protease inhibitor (Pierce) and 1.8× Halt phosphatase inhibitor (Pierce). Protein concentration was estimated using a Nanodrop spectrophotometer (Thermo, Wilmington DE), and 100 µg of protein was loaded into a 7.5% gel and subjected to SDS-PAGE at 80–120V for 1–2 hours. Proteins were transferred onto polyvinylidene fluoride membranes in a semi-dry transfer apparatus at 25V for 1 h, washed briefly, and membranes were blocked with 5% bovine serum albumin (BSA) for 1 hour at room temperature. Following a 5-minute wash, membranes were exposed for 1 hour at room temperature to anti-EphB4 primary antibody (anti-mouse; #265) or anti-ephrin-B2 primary antibody (anti-mouse; #2B5) at a concentration of 0.5 µg/mL in 2.5% BSA/0.05% Tween-20. Membranes were then washed three times for 10 minutes and incubated as above in anti-mouse secondary antibody conjugated to horseradish peroxidase (HRP) at a 1∶5000 dilution for 1 hour. Following another wash, membranes were incubated in enhanced chemiluminescence solution (Bio-Rad, Hercules CA) and photographed using a Bio-Rad QuantityOne imaging system. β-actin was probed similarly as a loading control. The prostate cancer cell line PC3 was used as a positive control for EphB4 expression [19].

### Flow Cytometry

Cells were washed once with phosphate buffered saline (PBS) and then dissociated with PBS/0.2% EDTA at 37°C for about 5 minutes. After neutralization of EDTA with culture media, cells were centrifuged at 1000rpm for 5 minutes. Cells were then washed with cold PBS/0.5%BSA, followed by incubation with 10% normal goat serum on ice for 30 m, and then with primary antibodies diluted in normal goat serum on ice for 1 hour. Cells were subsequently washed with cold PBS and incubated with fluorochrome-conjugated secondary antibodies on ice for 30 minutes. After a final wash with cold PBS, cell nuclei were stained with DAPI and analyzed with a flow cytometer (LSR II, BD Biosciences, Franklin Lakes NJ).

### Quantitative PCR

In order to determine EPHB4 gene copy number in tissue DNA, real-time quantitative PCR was performed using an Applied Biosystems StepOne Plus instrument (Foster City CA). Reaction mixtures contained Fast SYBR Green Master Mix (2X; Applied Biosystems), genomic DNA, forward and reverse qPCR primers (shown in Table S1), and molecular biology-grade water to total 25 µL per reaction. qPCR using LINE-1 primers was performed in parallel to serve as a gene copy number reference against which to normalize raw fluorescence values, and reactions containing various dilutions of control genomic DNA (Promega, Madison WI) were used to construct a standard curve to extrapolate tissue DNA concentrations and verify that these concentrations fell within the linear detection range of the instrument.

### siRNA Knockdown

For non-small cell lung cancer (NSCLC) cell lines, cells were plated using antibiotic-free medium in 96-well plates at a density of 2.0×10^4^ cells per well (for cell viability assays; eight or more replicates per experiment) or 6-well plates at a density of 5.0×10^5^ cells per well (for whole-cell lysate collection) and allowed to grow to 30–50% confluence. Cells were transfected using Oligofectamine transfection reagent (Life Technologies, Carlsbad CA) according to its standard protocol. FBS was added to 10% final concentration after 4 hours. Untransfected and mock transfected cells served as controls. Protein knockdown was confirmed by immunoblotting (Figure S3).

For small cell lung cancer (SCLC) cell lines, cells were plated using Opti-MEM media in 6-well plates at a density of 5×10^5^ cells per well (three replicates per condition). Cells were transfected with 100nM non-targeting control siRNA or EPHB4-targeting siRNA (Santa Cruz) using Oligofectamine transfection reagent according to its standard protocol. After incubation overnight, cells were collected by centrifugation and resuspended in complete media. Mock-transfected and control siRNA-transfected cells served as controls. Protein knockdown was confirmed by immunoblotting.

### Expression of EPHB4 Constructs in Cell Lines

A wild-type EPHB4 cDNA clone in the pCMV6-XL6 vector (Origene, Rockville MD) was used as a mammalian expression vector. For transient transfection, cells were plated in antibiotic-free medium in either 96-well plates at a density of 2.0×10^4^ cells per well (for cell viability assays; eight replicates per experiment) or 10-cm dishes at a density of 3.0×10^6^ cells per plate (for whole-cell lysate collection) and allowed to grow to approximately 50–75% confluence (to allow for exponential growth over the following 72 hours). Cells were transfected using Lipofectamine 2000 transfection reagent (Life Technologies) according to its standard protocol. Complexes were removed after 4–6 hours and replaced with fresh antibiotic-free medium after washing with PBS. Untransfected and mock-transfected (transfection reagent only) cells served as controls. Protein expression was confirmed by immunoblotting (Figure S4).

### Establishment of Stable EphB4-expressing Cell Lines

Wild-type full-length EPHB4 cDNA (GenBank accession number NM_004444; OriGene, Rockville MD) was cloned into pcDNA3.1 with a C-terminal Myc tag in frame. This vector and the control pcDNA3.1 empty vector were individually transfected into H661 cells using BioT (Bioland, Paramount CA) according the manufacturer’s protocol. Following transfection, the culture medium was changed to the final growth medium (RPMI1640 supplemented with 10% FBS) containing 300 µg/mL G418. One week later, surviving cells were trypsinized and plated on 96-well plates with graded dilutions. The concentration of G418 in the culture medium was reduced to 200 µg/mL from this point on. Single colonies emerged two weeks later and were screened by immunoblotting using an anti-Myc antibody (clone 9E10, ATCC). Colonies with exogenous EphB4-Myc expression were then expanded.

### Cell Viability Assays

For NSCLC cell lines, following transfection of siRNA or plasmids into cells or treatment of cells with sEphB4-HSA in 96-well plates as described above, cells were left to grow until the desired time point, at which time the media was removed, cells were washed once with PBS, and 100 µL fresh growth medium was added to each well. Following the addition of 5 µL of a 0.028% resazurin sodium salt solution (weight/volume; Sigma), plates were incubated at 37°C protected from light for 2–5 hours and fluorescence was measured using a fluorescence plate reader (530/590nm excitation/emission). For ephrin-B2/Fc stimulation assays, 5×10^4^ cells/well were seeded in 24-well plates, starved overnight, and stimulated with 1 µg/ml ephrin-B2/Fc for 0, 24, 48 or 72 hours. Cells were washed twice with 1× PBS, stained with Trypan Blue solution (0.4%; Sigma), and manually counted by light microscopy.

For SCLC cell lines, cells were plated in triplicate at a density of 1×10^5^ cells/well and treated with 0.5 µg/ml of ephrin-B2/Fc or the indicated concentrations of AZ12489875-002. For siRNA-transfected cells, cells were harvested 24 hours after transfection, resuspended in complete media, plated in triplicate at a density of 1×10^5^ cells/well, and left untreated or treated with 200nM of SN-38 for 72 hours at 37°C. For ephrin-B2/Fc stimulation assays, 5×10^4^ cells/well were seeded in 24-well plates, starved overnight, and stimulated with 0.5 µg/ml ephrin-B2/Fc for 48 hours. Cell viability was determined by adding 10 µL 5ng/ml MTT (3-(4,5-dimethylthiazol-2-yl)-2,5-diphenyltetrazolium bromide; Sigma) for the final 2–4 hours of culture. Insoluble formazan salts were dissolved by adding 50 µL of an acidified isopropanol solution. The absorbance was read at 570nm within 15 minutes of stopping the reaction using an absorbance plate reader.

### Topoisomerase I Activity Assay

H446 and H526 cells were left unstimulated or stimulated for 15 minutes with 50ng/ml of HGF or ephrin-B2/Fc, then washed twice with ice-cold PBS. Nuclear extracts were prepared as previously described [31]. Briefly, cells were collected by centrifugation and washed once with ice-cold TEMP (10 mM Tris-HCl (pH 7.5), 1 mM EDTA, 4 mM MgCl_2_, 0.5 mM phenylmethylsulfonyl fluoride (PMSF)). Cells were subsequently suspended in 1 mL of cold TEMP for 10 minutes and then centrifuged at 1500×*g* for 10 minutes. Nuclear pellets were resuspended in 20 µL TEP (10 mM Tris-HCl (pH 7.5), 1 mM EDTA, 0.5 mM PMSF) plus 20 µL 1 M NaCl, placed on ice for at least 30 m, and then centrifuged at 15,000×*g* for 15 minutes. *Top1* enzymatic activity in nuclear extracts was measured using a DNA relaxation assay (TopoGen, Port Orange FL) according to the manufacturer’s instructions. Briefly, 100ng of supercoiled plasmid DNA in a 20-µl reaction mixture (with 10 mM Tris-HCl (pH 7.9), 1 mM EDTA, 150 mM NaCl, 0.1% BSA, 0.1 mM spermidine, 5% glycerol) was incubated at 37°C for 30 minutes with neat and serially diluted (1∶4) nuclear extracts, purified recombinant human *Top1* (positive control), or assay diluent (negative control). Reactions were terminated by addition of 5 µl 5× loading buffer (5% SDS, 0.3% bromophenol blue). Samples were resolved on a 1% agarose gel and imaged as above.

### Soft Agar Colony Formation Assay

To evaluate the tumorigenic potential of the wild-type EphB4 cells, 1×10^4^ viable cells per well were plated in soft agar on 6-well plates. Briefly, the base layer was made by mixing equal volumes of sterile 1.2% agar (cooled to 40°C) and 2× RPMI1640 medium to obtain a final solution of 0.6% agar in 1× RPMI1640 medium. For the top layer, the agar was diluted to 0.8% in distilled water, cooled to 40°C, and then mixed in equal proportions with 2× RPMI1640 medium. The cells were immediately added to the mix to yield a final solution of 0.4% agar in 1× RPMI1640 medium. The cells grew for 4 weeks at 37°C in a humidified atmosphere containing 5% CO_2_, at which point viable colonies were photographed and counted using ImageJ software.

### Wound Healing Assays

H661 empty-vector and wild-type EPHB4 stable clone cells were seeded in 6-well plates and cultured until 100% confluent, then treated with 1 µg/ml ephrin-B2/Fc. A straight scratch was made across the cell layer using a 1-mL pipette tip. The cells were then gently washed with 1× PBS to remove cellular debris, and the media was replaced. Photographs were taken of the wound region at 0, 4, 7, 23, and 28 hours and analyzed by TScratch software (CSELab, ETH Zurich, Switzerland).

### *In vivo* Tumor Growth

2×10^6^ A549 or H1993 or 4×10^6^ H446 cells were injected into the flanks of 10–12-week-old male Balb/C athymic mice (Harlan Laboratories, Placentia CA) and allowed to establish primary tumors approximately 75 mm^3^ in volume. Flank injections were chosen over an orthotopic model due to their well-established use in lung cancer studies as well as their ease of non-invasive tumor measurements [32]. Tumor growth was measured thrice weekly, and volume was calculated using 0.52×a×b^2^ (derived from the volume calculation of a spheroid, V = 4/3 · π · a^2^ · b, where a is the radius of minor axis and b is the radius of the major axis; Ref. 33), where a is the longest dimension and b is the shortest dimension of the palpable mass. Six days after implantation, mice with A549 xenografts were divided randomly into four groups (six mice per group), and treatment was initiated: PBS (control), paclitaxel (20 mg/kg weekly), sEphB4-HSA (20 mg/kg thrice per week), or paclitaxel plus sEphB4-HSA. Mice with H1993 or H446 xenografts were divided into two groups (six mice per group), and treatment was initiated: PBS (control) or sEphB4-HSA (50 mg/kg thrice per week). All treatments were administered intraperitoneally. Animals were eventually sacrificed and tumors were harvested at the end of the experiment.

### Immunofluorescence

Tissues harvested from mouse A549 xenografts were snap frozen and embedded in OCT compound. 5–10 µm sections were fixed in 4% paraformaldehyde, washed in PBS, and incubated in primary anti-Ki-67 (BD Biosciences), anti-CD31 (BD Biosciences), anti-phosphorylated S6 (S235/S236; Cell Signaling, Danvers MA), anti-phosphorylated Akt [S473 (Ref. 34); Cell Signaling], or anti-phosphorylated Src (Y416; Cell Signaling) antibody overnight at 4°C. For immunofluorescence, sections were washed in PBS and incubated with Alexa Fluor-conjugated secondary antibody (Life Technologies). A TdT-mediated dUTP nick-end labeling (TUNEL) assay (Promega, Madison WI) was also performed to assess apoptosis. DAPI was used as a nuclear counterstain and served as a control against which cellular marker intensities were normalized. Images were captured with a Nikon Eclipse 80i fluorescence microscope and the Meta Morph imaging series system. For immunohistochemistry, sections were first incubated in biotin-conjugated secondary antibody, followed by HRP-conjugated avidin, then 3,3′-diaminobenzidine reagent (Vector Labs, Burlingame CA). Hematoxylin was used as a nuclear counterstain. Images were captured using an Olympus BX51 microscope and the Image-Pro Plus 6.0 system.

### Statistics

For immunohistochemical staining analyses, log_10_ ACIS values for tumor versus matched normal lung tissues were compared using a two-tailed paired t-test for the overall patient cohort as well as within individual subtypes. Each ACIS intensity score represents the mean of up to three replicates for a given patient determined using separate tissue cores within the same tumor microarray. The degree of similarity between ACIS and manual pathological scoring was determined using a two-tailed Spearman correlation. Survival data are represented using Kaplan-Meier curves based on immunohistochemical intensity scoring (high versus low using a cutoff score of 500 on ACIS, which represents the median overall intensity) and on race designation.

For cell viability assays, replicate data points were averaged and compared either to time-matched mock-transfected cells (for siRNA-transfected cells) or to time-matched control cells (for sEphB4-HSA-treated cells). To compare treatments pairwise, a student’s t test was used. Other comparisons of two groups were made using a student’s t-test, and those of three or more groups were made using one-way ANOVA. To assess variability between a set of treatment conditions with multiple time points, two-way ANOVA was used. Unless otherwise indicated, error bars used in displaying cell viability data represent standard error of the mean normalized to percent difference versus control values. For *in vivo* growth assays, the statistical significance of differences in tumor growth at each time point for each condition was determined using either a student’s t test for unpaired samples or one-way ANOVA for samples among four treatment groups. For determining the statistical significance of a change in mean tumor volume for an individual treatment arm, a student’s t test was used. All statistical calculations were performed using Prism software (GraphPad, La Jolla CA) or the SAS statistical package (Cary NC).

## Results

### EphB4 is Overexpressed and has Increased Gene Copy Numbers in Lung Cancer

**Figure 1:**
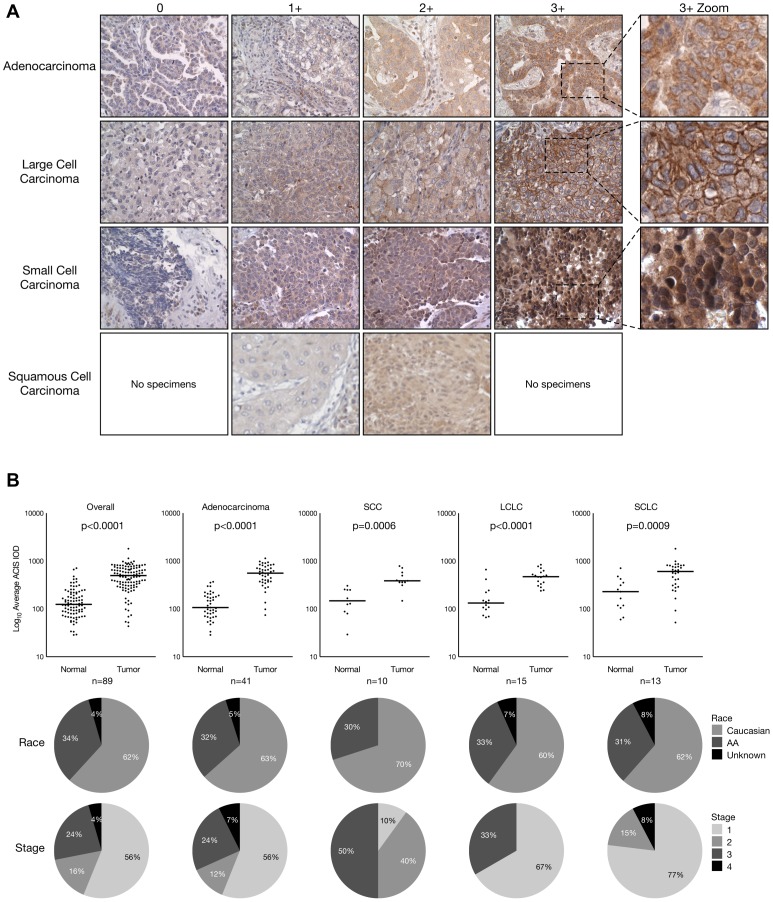
EphB4 protein expression in human lung cancer tissues. *A*. Representative immunohistochemistry images of EphB4 expression across multiple lung cancer subtypes. Pathological scoring is indicated above columns; rightmost panel is a higher- power view of corresponding 3+ images displaying subcellular staining patterns. Note pronounced membranous staining in large cell carcinoma. *B*. EphB4 expression patterns in various lung cancer subtypes with race and stage distribution. Individual points represent mean ACIS scores in a single patient for corresponding normal and tumor tissues. The total number of patients within each analysis is displayed below the graphs. Only patients with paired tumor and normal tissues were included in the analyses. Horizontal bars represent mean ACIS scores across all patients in a given set. Race and clinical stage distributions for each subtype and the overall set are displayed below each graph.

In pairwise analyses of 89 matched normal and tumor samples from lung cancer patients, we found EphB4 to be significantly overexpressed compared to paired normal tissues in adenocarcinoma (n = 41; 4.3-fold mean difference), large cell carcinoma (n = 15; 2.9-fold mean difference), small cell carcinoma (n = 13; 2.4-fold mean difference), and squamous cell carcinoma (n = 10; 2.7-fold mean difference) subtypes. Overall, tumors were found to express EphB4 3.2-fold more strongly than paired normal tissues (Figure 1). EphB4 in adjacent normal lung tissue is shown in Figure S5.

**Figure 2:**
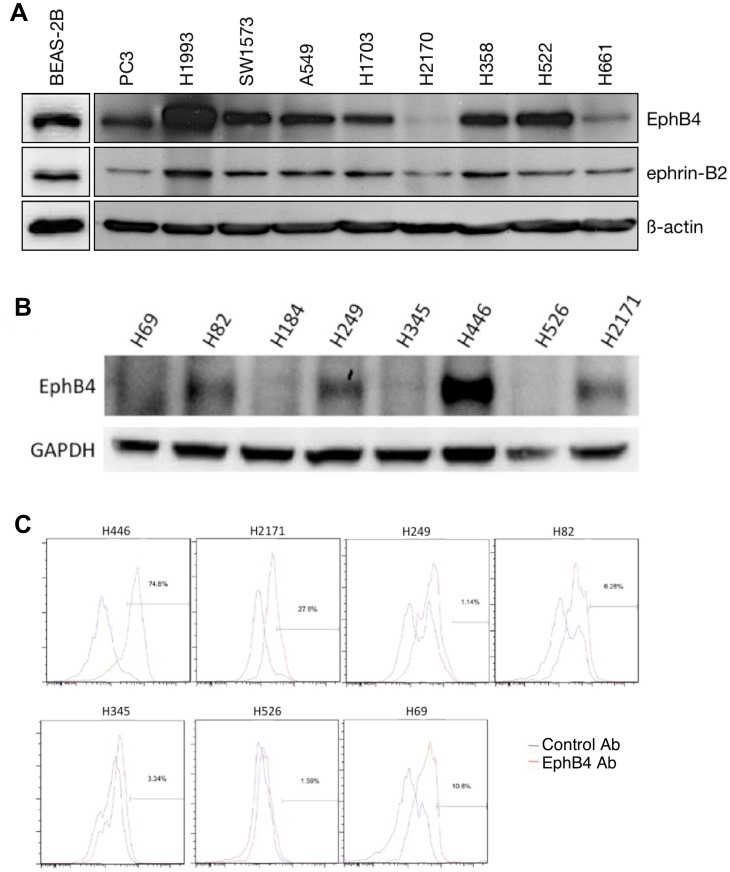
EphB4 protein expression in human lung cancer cell lines. *A–B*. EphB4 protein expression in panels of NSCLC and SCLC cell lines by immunoblotting. Ephrin-B2 expression was also assessed in NSCLC cell lines. BEAS-2B is an immortalized, non-cancerous lung cell line used here as a control; the prostate cancer cell line PC3 was included as a positive control. B-actin and GAPDH were used as loading controls. *C*. EphB4 expression in a panel of SCLC cell lines assessed by flow cytometry using EphB4-specific antibody (red). Normal mouse IgG was used as negative control (blue). EphB4 positivity expressed as the percentage of total cells is shown on the right of each panel.

We also determined NSCLC cell line expression of EphB4 protein via immunoblot analysis (Figure 2A). Six of eight NSCLC cell lines (A549, H358, H522, H1703, H1993, SW1573) expressed EphB4 to a substantial extent, one (H661) demonstrated low EphB4 expression, and one (H2170) lacked EphB4 expression. The EphB4 ligand ephrin-B2 was expressed at a consistently low level in all cell lines tested. Expression of EphB4 in a panel of eight SCLC cell lines was examined by immunoblotting (Figure 2B) and flow cytometry (Figure 2C). Four SCLC cell lines (H82, H249, H446, H2171) expressed EphB4 by immunoblot analysis, while H69 and H526 cells did not express EphB4. Surface expression of EphB4 by flow cytometric analysis largely validated the immunoblot findings.

To determine EPHB4 gene copy number in tissue samples, we conducted qPCR analysis and found that several tissues demonstrated increased EPHB4 gene copy numbers. Six, nine, and 23 percent of adenocarcinoma, small cell carcinoma, and squamous cell carcinoma tissues, respectively, were found to contain more than three EPHB4 gene copies, with 14% of squamous cell carcinoma tissues having more than 10 copies (Figure S6). No cell lines tested were found to demonstrate gene copy number gains.

### EphB4 Expression and Prognostication in Lung Cancer

**Figure 3:**
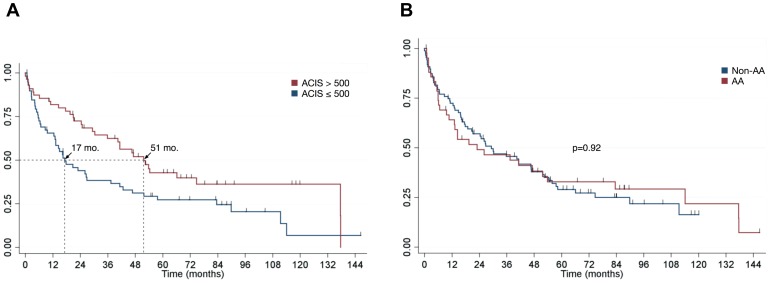
Kaplan-Meier analysis of patient survival. The ordinate axis in each graph represents the fraction of living patients. *A*. Survival stratified by EphB4 expression. ACIS scores above and below 500 were chosen for stratification based on the median score of the overall cohort. Arrows represent median survival (50% living patients) in months. *B*. Survival stratified by race.

A summary of clinical characteristics of patient tissues used in this study is shown in Table S2. Patient survival was strongly positively correlated with tumor expression of EphB4, as those with high EphB4 expression have a median survival threefold longer than those with low expression (51 months versus 17 months, p = 0.021; Figure 3A). Caucasian patients were also found to express EphB4 significantly more strongly than African-American patients (p = 0.019; data not shown); however, this did not translate into a difference in survival stratified by race (p = 0.92; Figure 3B). There was no significant association between EphB4 expression and other patient characteristics, such as gender, smoking status, and age at diagnosis, either in the overall patient cohort or when stratified by lung cancer subtype.

### EphB4 Modulation Affects Cell Growth *in vitro*

**Figure 4:**
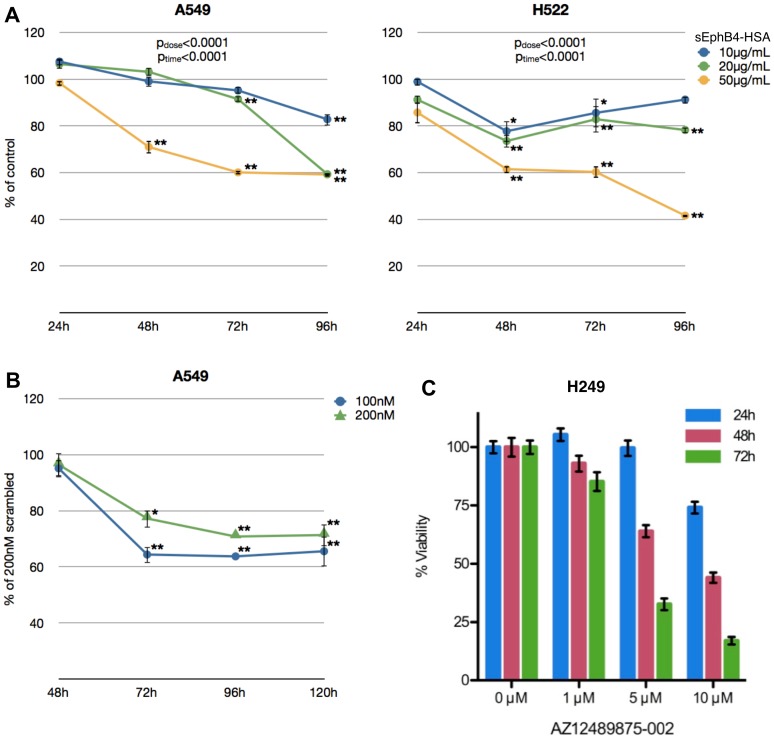
Reduced cell viability with EphB4 inhibition or knockdown. *A*. A549 and H522 cells were treated with soluble EphB4 at the concentrations indicated, and its effect on cell viability was measured over the time points shown using resazurin fluorescence. Cells treated with 50 µg/mL human albumin served as a control, and data points represent the percent versus matched control values at each time point. Each condition was repeated in eight replicates. Error bars indicate SEM. *, p<0.01; **, p<0.001 compared to time-matched control by student’s t test; overall p values shown were calculated by two-way ANOVA for time and dose. *B*. A549 cells were transfected with 100nM or 200nM EphB4-directed siRNA or a scrambled siRNA (sequences shown in Table S1), and their effect on cell viability was measured over the time points shown. Data points represent the percent versus matched scrambled siRNA values at each time point. Each condition was repeated in eight replicates. *, p<0.01; **, p<0.001 compared to scrambled siRNA. Error bars indicate SEM. *C*. H249 cells treated with the small-molecule inhibitor AS12489875-002 at varying concentrations were analyzed for cell viability at the time points shown. Error bars indicate SD.

Lung cancer cells were transiently transfected with EPHB4-directed siRNA or treated with sEphB4-HSA in culture to determine whether cell viability would be affected. sEphB4-HSA is a monomeric protein fragment comprising the extracellular domain of EphB4 and conjugated to human serum albumin that can bind ephrin-B2 ligand and therefore antagonize EphB4 receptor activation through disruption of the interaction between EphB4 receptor and ephrin-B2 ligand [35], [36]. In the A549 cell line, viability after exposure to sEphB4-HSA was reduced by approximately 17% with the lowest concentration and by 40% with the highest concentrations of sEphB4-HSA after 96 hours (Figure 4A). In the H522 cell line, a similar effect was seen in the highest dose after 96 hours (∼58% reduction), while the lower two concentrations initially reduced viability compared to control cells but had a diminished effect at later time points (Figure 4A).

In the A549 cell line, a statistically significant reduction in cell viability was observed in cells transfected with EPHB4-directed siRNA compared to scrambled siRNA-transfected cells (Figure 4B), resembling the inhibition profile using sEphB4-HSA. While the 100nM siRNA exposure resulted in a greater reduction (∼35%) in cell viability than the 200nM concentration (∼29%), these were not significantly different from each other at any time point. Ephrin-B2 expression did not change after EphB4 knockdown (data not shown). We also explored the effect of EphB4 inhibition in SCLC using the small molecule inhibitor AZ12489875-002. In H249 cells, EphB4 inhibition by AZ12489875-002 decreased cell viability in a dose-dependent manner (Figure 4C).

**Figure 5:**
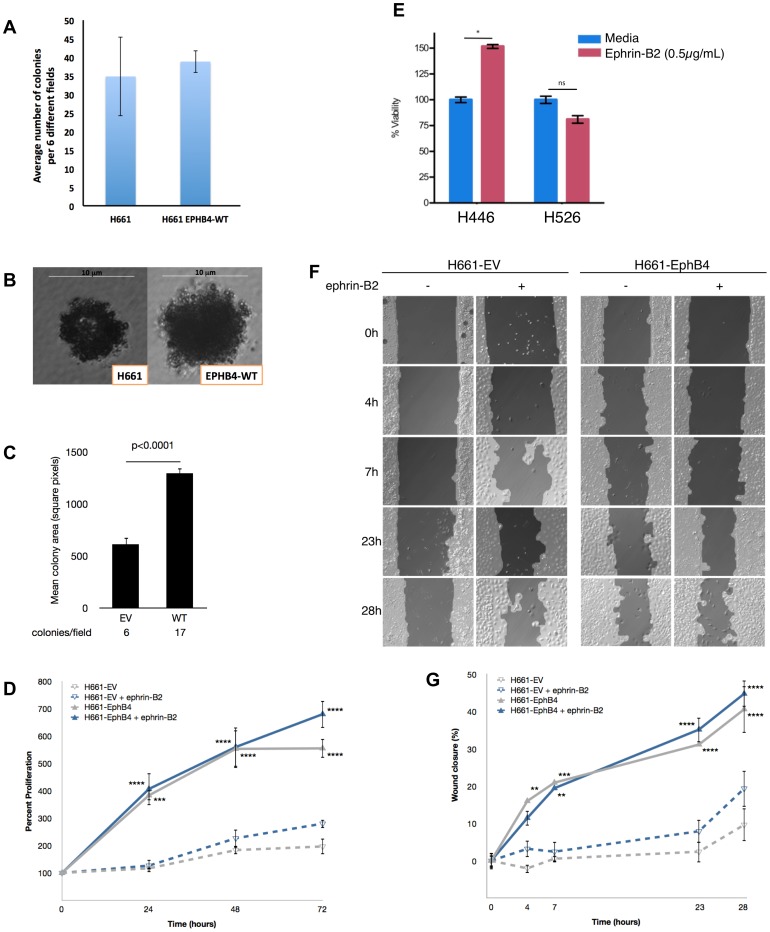
EphB4 modulation affects cell growth and migration *in vitro*. *A*. H661 cells were transfected with either wild-type EphB4 empty-vector constructs, and their effect on cell growth was assessed with or without stimulation with ephrin-B2/Fc. The number of colonies formed in EV-transfected and EphB4-transfected stable clones is shown. Each condition was repeated in six replicates. Error bars indicate SEM. *B*. Representative colonies as assessed in Panel *A*. *C*. Quantification of average colony size of EphB4-harboring H661 cells versus control cells. Error bars indicate SEM. *D*. Cell proliferation with and without ephrin-B2 stimulation is shown. Data are represented as mean percent change relative to the initial time point among three replicates. Error bars indicate SEM normalized to percent proliferation. ***, p<0.001; ****, p<0.0001 relative to H661-EV. *E.* H446 and H526 cells were stimulated with ephrin-B2 ligand and assessed for cell viability. *, p<0.01; ns, not significant. *F*. Representative images of wounds created in confluent layers of cells in culture. *G*. Quantification of wound closure. Data are represented as average percent wound closure compared to the initial wound size among six independent replicates. Error bars indicate SEM normalized to percent closure. **, p<0.01; ***, p<0.001; ****, p<0.0001 relative to H661-EV.

The H661 cell line was used as a model in which to test the effects of exogenous expression of wild-type EphB4. Stable expression of wild-type EphB4 resulted in a 16% increase in cell proliferation after 24 hours compared to mock-transfected cells (p = 0.0001; data not shown), although this gain of function was reduced to a 9% increase after 48 hours (p = 0.5314). These data suggest that wild-type EphB4 may provide some stimulation for cell proliferation. Using a soft agar colony formation assay, it was observed that H661 cells stably transfected with wild-type EphB4 demonstrated a small, statistically insignificant increase in colony formation over 4 weeks relative to mock-transfected control cells (Figure 5A); however, colonies formed from EphB4-harboring cells were over two-fold larger than colonies formed in control cells (p<0.001; Figure 5B–C). Transfection of H661 cells with EphB4 resulted in a significant increase in cellular proliferation over 72 hours (p<0.0001; Figure 5D). When EV- and wild-type EphB4-transfected cells were treated with clustered ephrin-B2/Fc, the degree of increased proliferation was similar to transfection with wild-type EphB4 alone. However, there was no statistically significant increase in proliferation in cells treated with ephrin-B2/Fc compared to non-treated cells (Figure 5D), suggesting that the effects of EphB4 are ligand-independent.

To determine whether EphB4/ephrin-B2 signaling could enhance cellular proliferation in SCLC cell lines, we measured the viability of an EphB4-positive cell line (H446) and an EphB4-negative cell line (H526) following stimulation with ephrin-B2/Fc. In H446 cells, ephrin-B2/Fc induced a significant increase in cellular proliferation over 48 hours compared to cells maintained in complete medium (p<0.01), while the H526 cell line was unresponsive to ephrin-B2/Fc stimulation (Figure 5E).

### EphB4 Enhances Cellular Migration

We used the H661 cell line to additionally test the effects of EphB4 on directional cell migration using wound healing assays. Compared to mock-transfected controls, H661 cells stably transfected with wild-type EphB4 exhibited increased migration as determined by percent wound closure (40.6% wound closure in EphB4-harboring cells after 28 hours versus 9.6% in control cells; p<0.0001; Figure 5F–G). Degree of wound closure was similar after stimulation with ephrin-B2/Fc; however, there was no statistically significant increase compared to non-treated cells (Figure 5F–G), again suggesting that effects induced by EphB4 expression are independent of its ligand.

### siRNA-mediated EphB4 Silencing Decreases SCLC Cell Viability and is Enhanced in Combination with Topoisomerase Inhibition

**Figure 6:**
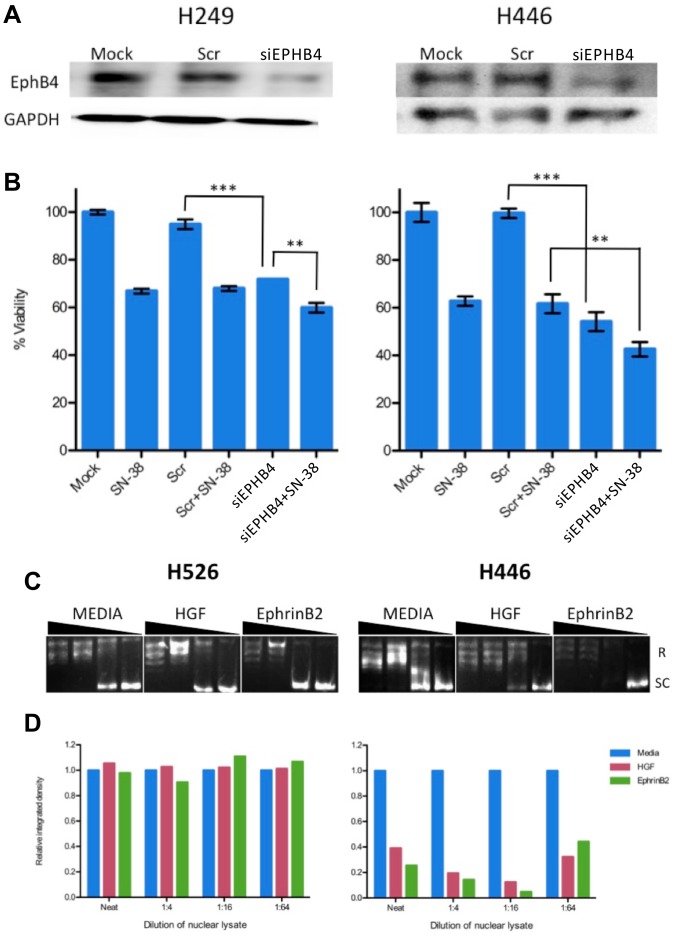
EphB4 protein knockdown reduces SCLC cell viability. *A.* EphB4 protein knockdown following siRNA transfection. GAPDH was used as a protein loading control. Scr, scrambled siRNA. *B.* Viability of cells following EphB4 knockdown and/or treatment with SN-38. Error bars indicate SD. **, p<0.01; ***, p<0.001. *C.* Representative images of topoisomerase I relaxation assays. Cells were left unstimulated (media) or stimulated for 15 minutes with HGF or ephrin-B2, and nuclear lysates were harvested for use in topoisomerase I relaxation assays. Nuclear lysates were assayed undiluted (neat) or serially diluted as indicated. R, relaxed conformations of plasmid DNA; SC, supercoiled conformation of plasmid DNA. *D.* The relative integrated density of the SC band was quantified and normalized to media for each condition. Data shown are representative of two independent experiments.

siRNA-mediated knockdown of EphB4 in SCLC cell lines reduced EphB4 protein expression (Figure 6A) and resulted in a significant decrease in cell viability in the H446 (high EphB4 expression) and H249 (low EphB4 expression) cell lines (p<0.001; Figure 6B). Because topoisomerase I inhibition is a novel therapeutic approach in SCLC, particularly in refractory or relapsed disease [37], we next sought to determine whether topoisomerase I inhibition using SN-38 could enhance the observed reduction in cell viability. In H249 cells, knockdown of EphB4 reduced cell viability to a similar degree as treatment with SN-38, whereas the combination of EphB4 siRNA and SN-38 reduced cell viability to a greater extent than EphB4 siRNA alone (p<0.01), suggesting an additive effect. In H446, EphB4 knockdown was more effective than treatment with SN-38, while the combination of EphB4 siRNA and SN-38 reduced cell viability to a greater extent than EphB4 siRNA alone or SN-38 alone (p<0.01). The H446 cell line was more sensitive in general to combined inhibition of EphB4 and topoisomerase I than H249 (Figure 6B).

### EphB4/ephrin-B2 Stimulation Induces Topoisomerase I Activity in SCLC Cell Lines

Given the potential clinical promise for topoisomerase I inhibition in the context of SCLC and the observation that EphB4 knockdown in combination with topoisomerase I inhibition demonstrates additive reduction of cell viability, we examined the activity of topoisomerase I in response to stimulation of EphB4/ephrin-B2 signaling. Treatment of cells with ephrin-B2/Fc induced topoisomerase activity, as assessed by DNA relaxation, in cells with high expression of EphB4 (H446) but not in those with negative EphB4 expression (H526; Figures 6C–D).

### Inhibition of EphB4 *in vivo* reduces Volume of Established Tumors

**Figure 7:**
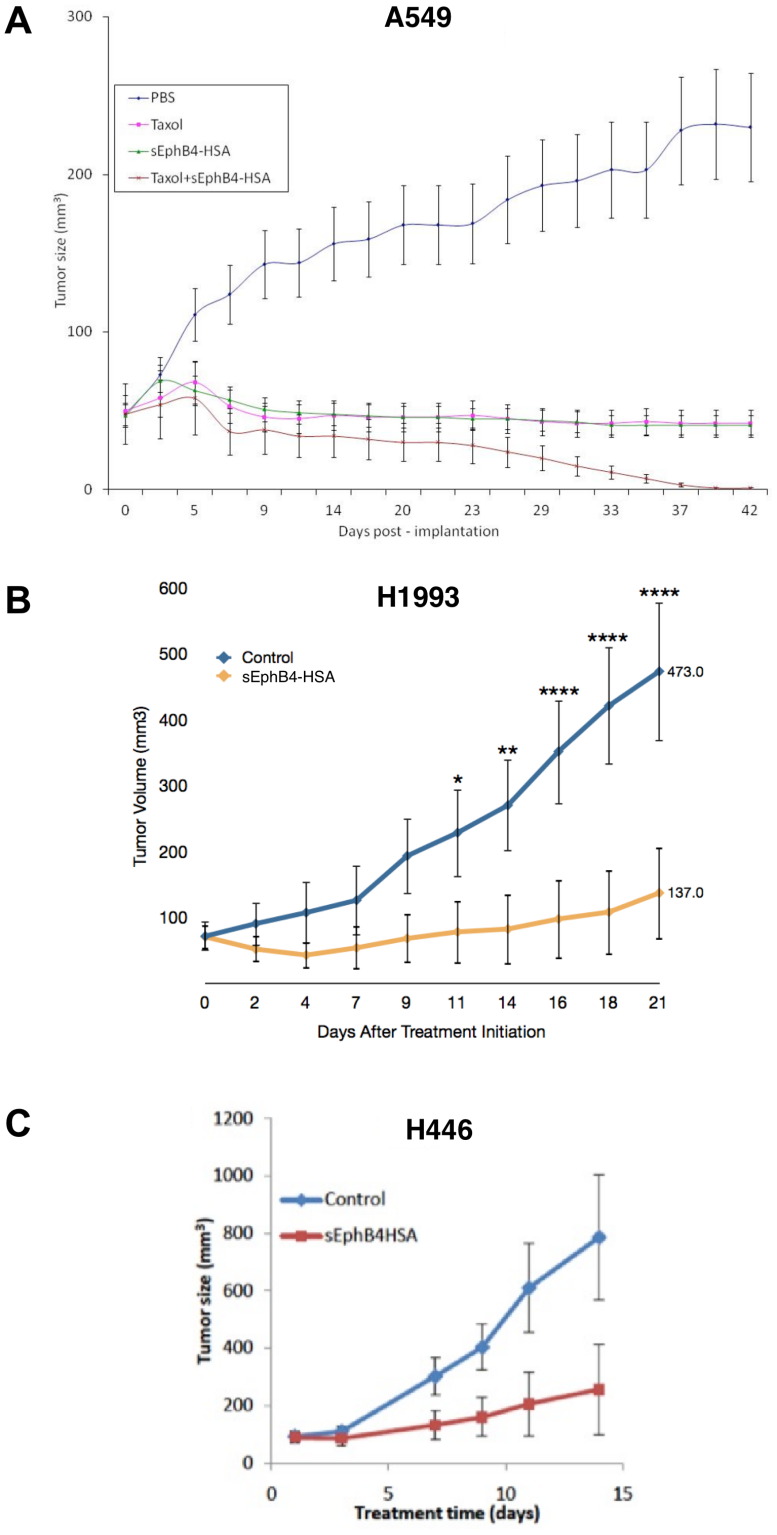
Tumor remission in mouse lung xenografts in response to EphB4 inhibition alone and in combination. *A*. A549 cells were injected into flanks of nude mice, allowed to establish primary tumors, and treated with PBS (control), paclitaxel, soluble EphB4 (sEphB4-HSA), or paclitaxel plus soluble EphB4. Error bars indicate standard deviation among six mice per group. *B–C*. H1993 or H446 cells were injected into flanks of nude mice, allowed to establish primary tumors, and treated with PBS (control) or soluble EphB4 (sEphB4-HSA). Error bars indicate SEM among six mice per group. *, p<0.05; **, p<0.01; ****, p<0.0001.

The effects of EphB4 inhibition with sEphB4-HSA were investigated using mouse tumor xenograft models. A549 lung tumor xenografts treated with paclitaxel or sEphB4-HSA as single agents had similar rates of growth to the control tumors initially following treatment but later regressed to the baseline tumor size 7–9 days later and remained at a constant size until Day 42, resulting in tumors 75–80% smaller compared to control mice (p = 0.0004 for paclitaxel versus PBS, p = 0.0003 for sEphB4-HSA versus PBS; Figure 7A). However, tumors treated with a combination of paclitaxel plus sEphB4-HSA exhibited a dramatic decrease in size, eventually reaching complete remission after approximately 39 days after the initiation of treatment (p = 0.0002 versus PBS; Figure 7A). A similar experiment carried out using H1993 xenografts demonstrated a 71% reduction of *in vivo* tumor growth within 21 days (p<0.0001; Figure 7B). In SCLC, sEphB4-HSA treatment inhibited the growth of xenograft tumors by 68% after 14 days (p<0.01; Figure 7C).

**Figure 8:**
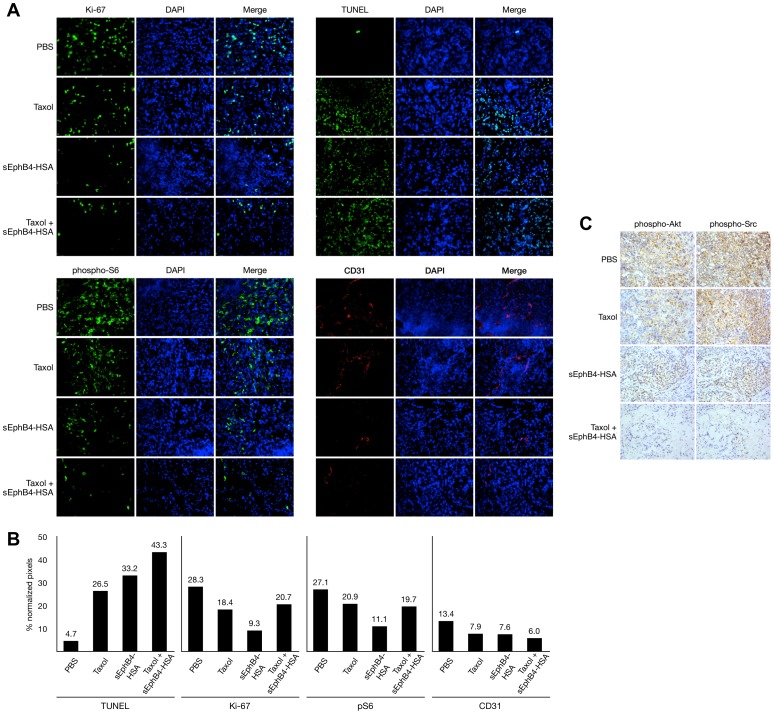
Expression of tumor-associated biomarkers in NSCLC tumor xenografts treated with sEphB4-HSA and paclitaxel *in vivo*. *A*. Ki-67 staining, TUNEL staining, phosphorylated S6 expression, and CD31 staining are demonstrated by immunofluorescence. DAPI was used as a nuclear counterstain. *B*. Quantification of fluorescence intensity normalized to DAPI. Values atop each bar represent percent intensity relative to the corresponding DAPI image. sEphB4-HSA, soluble EphB4; pS6, phosphorylated S6. *C*. Phosphorylated Akt and phosphorylated Src expression are demonstrated by immunohistochemistry.

At the conclusion of these studies, A549 and H446 tumor xenografts were harvested and examined for the expression of tumor-associated biomarkers. In response to single-agent or combination treatment of A549 xenograft tumors, TdT-mediated dUTP nick-end labeling (TUNEL) staining was increased, suggesting increased apoptotic activity among tumor cells, while Ki-67, phospho-S6, CD31, phospho-Akt, and phospho-Src signals were reduced, indicating reduced tumor cell proliferation, reduced neoangiogenesis, and reduced activity of downstream signaling pathways in treated cells (Figure 8A–C). H446 xenograft tumors treated with sEphB4-HSA exhibited decreased RCA-lectin, CD31, Ki-67, phospho-S6, and phospho-Akt staining, as well as increased caspase-3 staining, suggesting the induction of apoptosis (Figure S7).

## Discussion

The data reported here demonstrate that EphB4 is significantly overexpressed in lung cancer and its gene locus frequently demonstrates increased copy numbers in lung cancer. Additionally, we have shown that EphB4 is crucial for the growth of lung cancer cells *in vitro* and *in vivo* and that modulation of EphB4 protein expression has significant effects on the motility of lung cancer cells. Few other studies have previously investigated the role of EphB4 in lung cancer, and none have done so in such a systematic manner. Recently, Zheng et al. found that EphB4 is expressed more strongly in tumor tissues compared to paired normal samples and that expression was positively correlated with clinical stage [38]. However, there were relatively few samples in this study, and other parameters such as survival, effects of protein inhibition, and *in vivo* biology were not explored.

Overall, the pattern of overexpression observed here in lung tumors was remarkable, and it is perhaps related to the gene copy number findings, in which a subset of tumor tissues were found to have increased EPHB4 gene copy numbers. In particular, increased gene copies in adenocarcinoma and small cell lung cancer samples may underlie the protein overexpression noted in Figure 1. However, it is interesting that squamous cell carcinoma (SCC) featured the most prevalent degree of increase in gene copy number while having a relatively homogeneous expression profile. The aforementioned stage breakdown notwithstanding, another possible reason for this is simply that different tissues were used for each analysis; unfortunately, there was virtually no overlap of the 10 patient samples analyzed for protein expression with the 22 patient samples analyzed for gene copy number, so no definite connection can be made linking copy number increases with protein overexpression in SCC. It is also interesting that a significant difference in EphB4 was detected between Caucasians and African-Americans, although this did not translate into a difference in survival. It may be the case that our sample size was too small to detect such a difference, and it is certainly premature to make any conclusions about differential EphB4 biology among races based on these data alone.

A surprising finding was that EphB4 expression in lung cancer positively correlated with patient survival. It is typically the case that overexpression of RTKs in cancer leads to poorer survival. Indeed, MET [39] and EGFR [40] are both negatively correlated with survival in patients with lung cancer. It is thought that overexpression and activation of downstream pathways for each of these receptors leads to shortened survival as a result. In addition, EphA2 was recently shown to demonstrate a similar trend in lung cancer in which patients expressing low levels of EphA2 had a median survival that was over threefold longer than in patients with strong EphA2 expression [9]. However, the trend observed here with EphB4 is not without considerable precedent. Although they are typically regarded as having pro-tumor effects, expression of Bcl-2 and phosphorylated ERK1/2 in NSCLC patients receiving chemotherapy were positively correlated with recurrence-free survival [41]. Expression of CXCR4 in lung cancer has also been linked to longer overall survival and longer disease-free survival for total CXCR4 expression [42] and to longer overall survival for nuclear CXCR4 expression [43]. Recently, the EML4/ALK fusion protein has also been demonstrated to trend toward positive prognostication in lung cancer [44], [45]. Indeed, in colorectal cancer, while increased EphB4 expression is associated with longer patient survival [46], inhibition of EphB4 leads to decreased cell proliferation and metastasis and expression of EphB4 enhances tumor growth [24]. Postoperative survival of early-stage NSCLC patients was positively correlated with EphA2 mRNA expression [47] despite a preponderance of preclinical and clinical data demonstrating its oncogenic effects. It is based on these prior findings that EphB4 may also be considered a positive prognostic indicator in lung cancer given the data presented here. Elucidating why this relationship exists is a question that should be addressed in future studies; however, we have attempted to shed some light on it here based on prior observations within the receptor tyrosine kinase family.

Importantly, this relationship does not imply that EphB4 cannot or should not be targeted clinically; on the contrary, other pro-tumorigenic molecules with similar relationships to patient survival, such as the aforementioned Bcl-2 and EML4-ALK, have proven to be good targets for inhibition. It may therefore be the case that EphB4 expression suggests some susceptibility to chemotherapy, thus prolonging patient survival. There seems to be little consistency across Eph receptors and their established role in pro- or antitumorigenic effects. For example, Herath et al. recently showed that EphB4 is overexpressed in colon cancer relative to normal tissue [48]; however, other groups had previously shown that EphB4 expression is negatively correlated with tumor progression [49]. One possibility that could explain these discrepancies is the interface of Eph receptors with downstream networks that regulate tumorigenicity, such as that between EphB3 and the PP2A/RACK1/Akt axis in lung cancer [50], [51]. Such an investigation into protein-protein interaction networks involving EphB4 using a systems biology approach has not been conducted but is certainly warranted as it may shed some light on this paradox.

One potentially confounding factor is that the treatments that this cohort of patients underwent prior to investigation of EphB4 protein expression in their tissues is not fully known; therefore, an alternative explanation for our findings is that those who were treated with chemotherapy regimens or even small-molecule tyrosine kinase inhibition (TKI), neither of which are 100% specific to a single molecular target within tumor cells, survived longer simply because they expressed EphB4 and therefore EphB4 activity was partially being abrogated. In such a case, patients expressing higher levels of EphB4 would appear to survive longer since EphB4 was effectively, if inadvertently, being inhibited.

Topoisomerase inhibition has shown significant clinical promise in lung cancer, particularly in combination with other therapeutic agents (reviewed in Ref. 52). The data presented here are not the first data to demonstrate a link between RTK activity and subsequent topoisomerase upregulation. SCLC cells have been shown to exhibit decreased topoisomerase I activity following treatment with a c-Kit small molecule inhibitor [53]. Similarly, MET upregulates topoisomerase I in NSCLC, especially in the setting of resistance to EGFR TKI [54]. EphB4 appears to induce a similar response in topoisomerase activity, and it would therefore be interesting to investigate whether this relationship plays a significant role in parallel to EGFR- or MET-mediated mechanisms of TKI resistance.

The suppression of EphB4 in cultured cells was shown via three methods to reduce cell growth, suggesting that EphB4 is a critical cellular survival factor in lung cancer. Additionally, overexpression of EphB4 was demonstrated here to enhance directional migration *in vitro*. Although EphB4 has traditionally been considered to be primarily an effector of cytoskeletal rearrangement and therefore cellular motility, these data, taken together with the pro-proliferative findings, suggest that the primary roles of EphB4 in lung cancer cells may involve both cellular survival and movement. This is in agreement with earlier studies in mesothelioma cell lines in which knockdown of EphB4 was shown to reduce cellular motility and invasiveness in addition to proliferation [21]. More studies investigating the link between EphB4 and cytoskeletal dynamics in the lung are warranted, especially those exploring invasion, motility, and cellular morphology specifically.

The H249 SCLC cell line was found to be exquisitely sensitive to treatment with the AZ12489875-002 EphB4 small molecule inhibitor to an extent greater than with sEphB4-HSA or siRNA knockdown. There are a number of reasons why this discrepancy may be present. First, the cells treated with AZ12489875-002 were derived from SCLC tumors, while those treated with sEphB4-HSA or siRNA were derived from NSCLC tumors, and this may be the source of some fundamental differences in cellular biology among lung cancer subtypes, such as intrinsic differences between suspension cells (SCLC) versus adherent cells (NSCLC). Second, we additionally subjected H249 cells to EphB4-targeted siRNA knockdown and found that this alone was sufficient to significantly reduce cellular growth, suggesting that EphB4 expression is crucial to cellular viability in this cell line and that this particular cell line may be particularly sensitive to EphB4 modulation. Finally, it is possible that AZ12489875-002 is not specific to EphB4 but rather targets other RTKs with varying affinities. The original literature on the pharmacokinetics and specificities of EphB4 small molecule inhibitor development [55]–[58] has specifically investigated FGFR1 and VEGFR2 as potential targets of similar molecules. The specificity of most molecular variants for EphB4 is several orders of magnitude greater than for other RTKs, and we therefore feel confident that this agent is indeed relatively specific for EphB4 in our in vitro studies. However, this possibility as a cause for the discrepancy seen here cannot yet be definitively confirmed.

In accordance with the *in vitro* inhibition data presented here, EphB4 inhibition *in vivo* proved to be extraordinarily effective in limiting the growth of established tumors or, in combination with paclitaxel, causing near-complete or complete remission of established xenograft tumors. Additionally, treatment of mice harboring tumors altered expression of several tumor biomarkers, including markers of cell proliferation, apoptosis, tumor vasculature, and downstream signaling. Notably, CD31 expression on endothelial cells was shown to be an important prognostic factor in NSCLC in conjunction with nucleolin [59]. Importantly, soluble EphB4 used as a single agent was found to be just as effective in limiting xenograft growth in the A549 cell line as was paclitaxel, a non-targeted chemotherapeutic agent that is sometimes used in combination with other modalities and drugs, including other chemotherapies as well as targeted kinase inhibitors such as gefitinib [60]–[62]. sEphB4-HSA blocks the interaction between EphB4 receptor and ephrin-B2 ligand, thereby inhibiting EphB4 phosphorylation, activation, and downstream signaling. We demonstrate the *in vivo* consequences of this inhibition here through decreased phosphorylation of S6 kinase, Akt, and Src, which are known to be involved in signaling downstream of RTK activation to promote cellular growth within lung tumors. Taken together, the findings stemming from soluble EphB4 used *in vivo* strongly implicate EphB4 as an attractive target that should be explored further in the clinical setting. Clinical trials investigating the efficacy of single-agent EphB4 inhibition as well as combination therapy involving EphB4 inhibition may be warranted given these data.

## Supporting Information

Figure S1 **Correlation of ACIS and IHC expression analysis methods.** *A*. Overall patient cohort stratified by histology. *B*. Overall patient cohort stratified by clinical stage. Error bars indicate SEM. The two quantification methods had a correlation of r2 = 0.75, p<0.0001. Variation in tumor expression of EphB4 was statistically significant across subtypes (p = 0.0008; one-way ANOVA) and clinical stages (p = 0.0308; one-way ANOVA).
(TIFF)

Figure S2 **Representative immunohistochemical staining of fresh frozen and formalin-fixed paraffin-embedded tissue specimens.**
(TIFF)

Figure S3 **siRNA knockdown of EphB4 in cell lines.** A549 and H1993 cells were transfected with EPHB4-targeted siRNA over 72 h at the nanomolar concentrations shown. Values shown below blots represent band intensities compared to the control lane, set arbitrarily to a value of 1.00, and normalized to ß-actin. C, untransfected control cells; M, mock-transfected cells; Scr, cells transfected with 200nM scrambled siRNA.
(TIFF)

Figure S4 **Expression of EphB4 in a non-EphB4-expressing lung cancer cell line.** H661 was transfected with wild-type EphB4. Untransfected cells and cells treated with transfection reagent only served as negative controls.
(TIFF)

Figure S5 **EphB4 protein expression in adjacent normal human lung cancer tissues.** Representative immunohistochemistry images of EphB4 expression in normal tissue adjacent to tumor foci. Pathological scoring is indicated above images.
(TIFF)

Figure S6 **EPHB4 gene copy numbers in human lung cancer tissues and cell lines.** The number of tested samples within each subtype or source is denoted in parentheses.
(TIFF)

Figure S7 **Expression of tumor-associated biomarkers in SCLC tumor xenografts treated with soluble EphB4** ***in vivo*** **.** RCA-lectin, CD31, Ki-67, and caspase-3 staining are demonstrated by immunofluorescence. DAPI was used as a nuclear counterstain. Phosphorylated S6 and phosphorylated Akt expression are demonstrated by immunohistochemistry.
(TIFF)

Table S1 **Sequences of siRNA oligonucleotides used for gene knockdown.**
(TIFF)

Table S2 **Summary of patient characteristics corresponding to lung cancer tissues used for protein expression and survival analyses.**
(TIFF)
