# Supplementary figures and images for: The EphB4 Receptor Tyrosine Kinase Promotes Lung Cancer Growth: A Potential Novel Therapeutic Target
Source: PLoS One. 2013 Jul 2;8(7):e67668. doi: 10.1371/journal.pone.0067668 (PMC3699624; doi:10.1371/journal.pone.0067668)

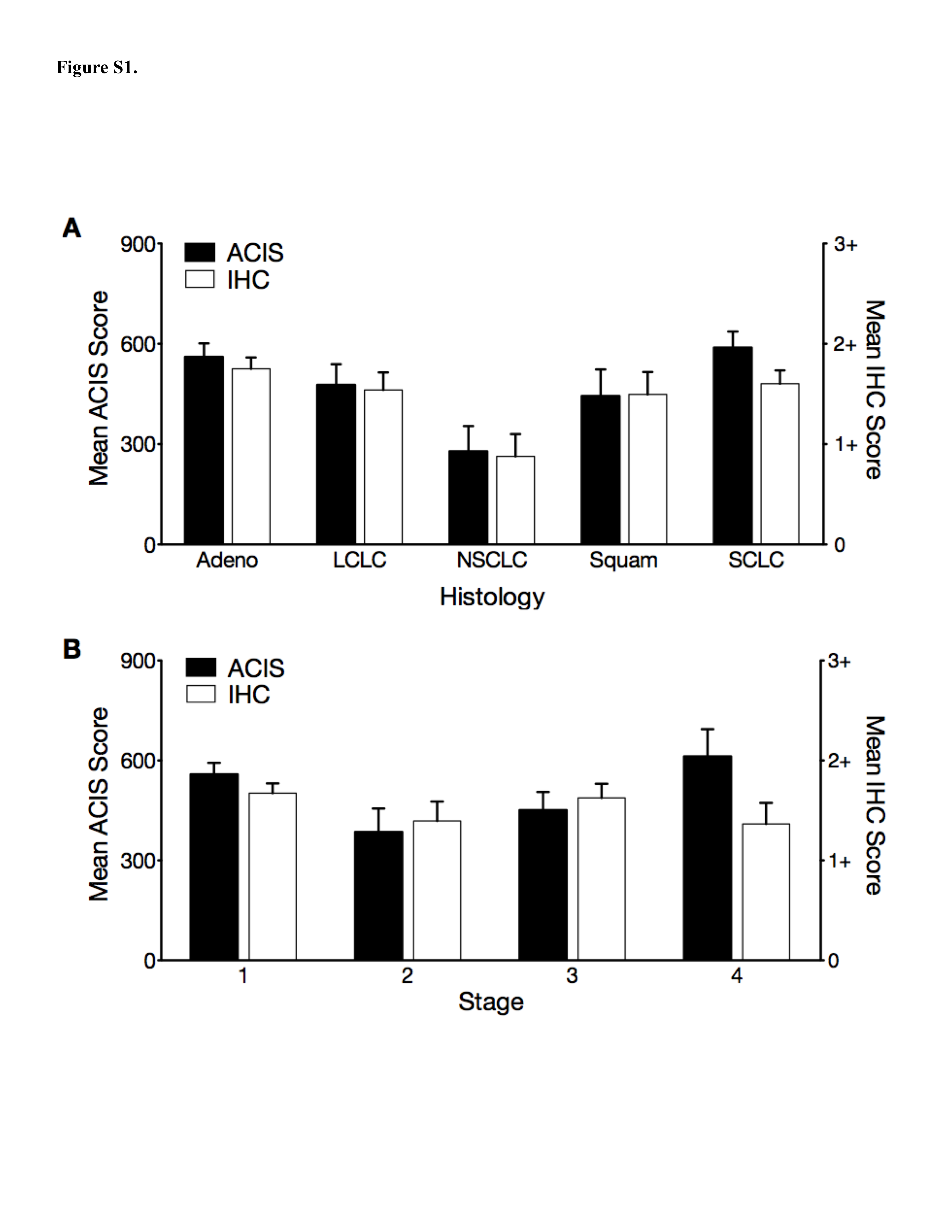

Supplement: Figure S1 — Correlation of ACIS and IHC expression analysis methods. A. Overall patient cohort stratified by histology. B. Overall patient cohort stratified by clinical stage. Error bars indicate SEM. The two quantification methods had a correlation of r2 = 0.75, p<0.0001. Variation in tumor expression of EphB4 was statistically significant across subtypes (p = 0.0008; one-way ANOVA) and clinical stages (p = 0.0308; one-way ANOVA). (TIFF) [file pone.0067668.s001.tiff]

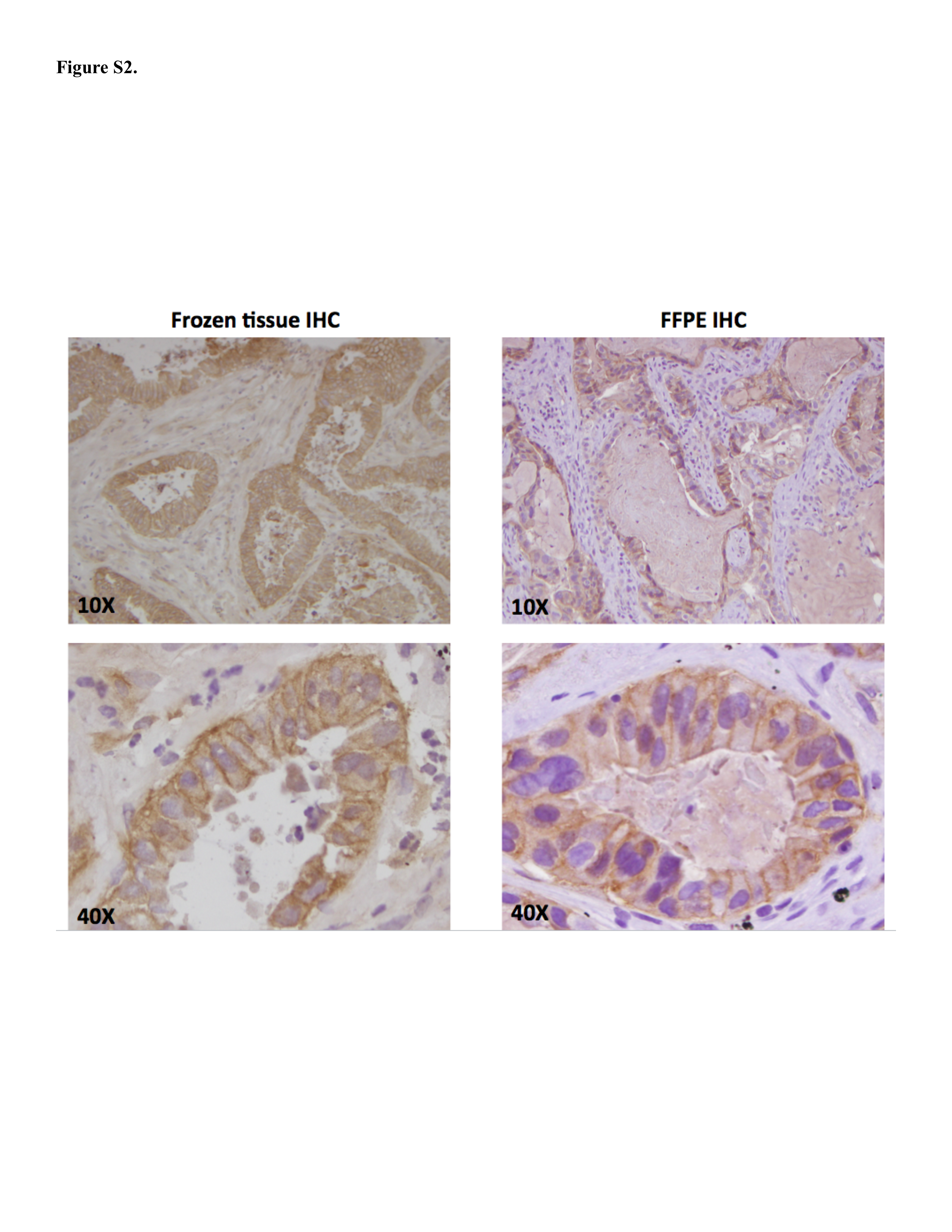

Supplement: Figure S2 — Representative immunohistochemical staining of fresh frozen and formalin-fixed paraffin-embedded tissue specimens. (TIFF) [file pone.0067668.s002.tiff]

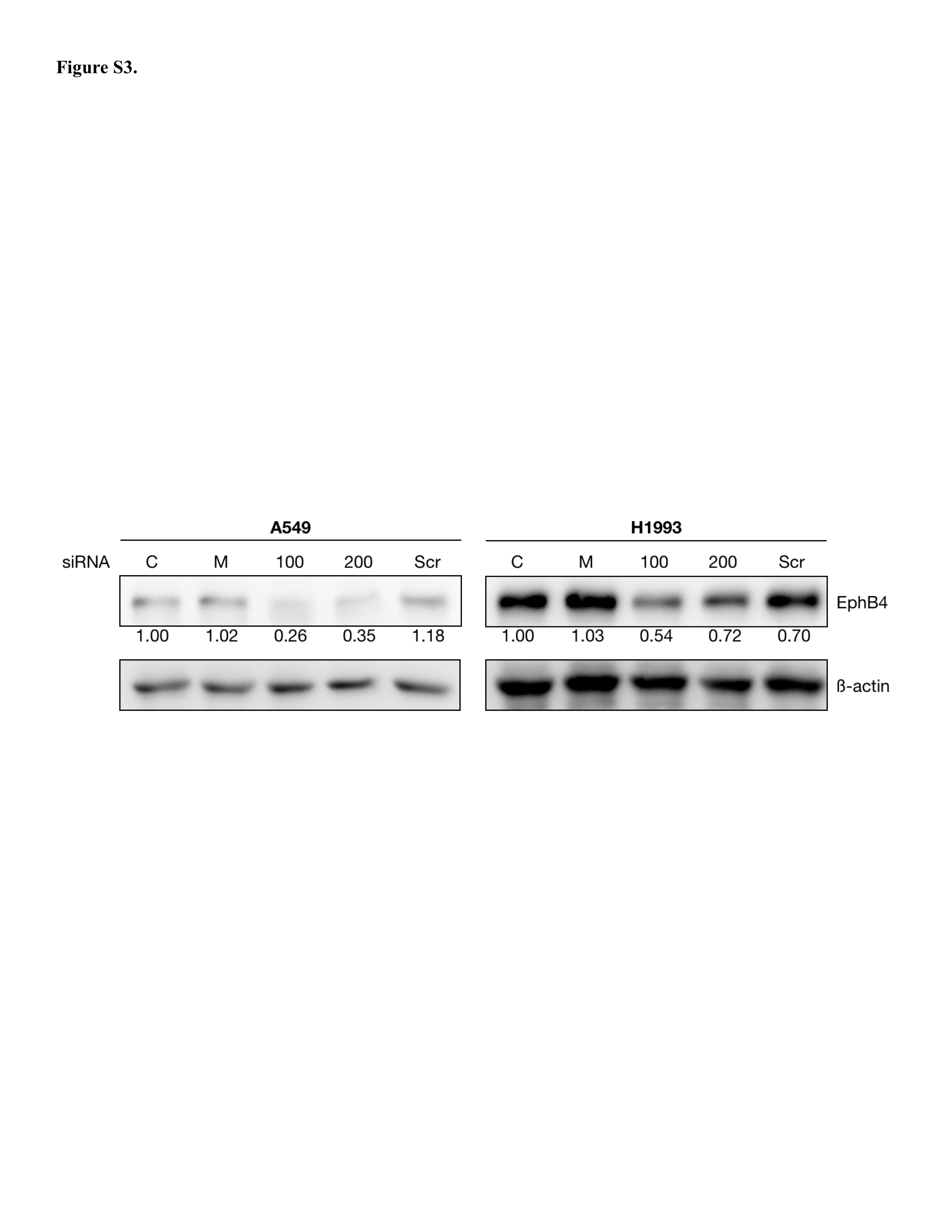

Supplement: Figure S3 — siRNA knockdown of EphB4 in cell lines. A549 and H1993 cells were transfected with EPHB4-targeted siRNA over 72 h at the nanomolar concentrations shown. Values shown below blots represent band intensities compared to the control lane, set arbitrarily to a value of 1.00, and normalized to ß-actin. C, untransfected control cells; M, mock-transfected cells; Scr, cells transfected with 200nM scrambled siRNA. (TIFF) [file pone.0067668.s003.tiff]

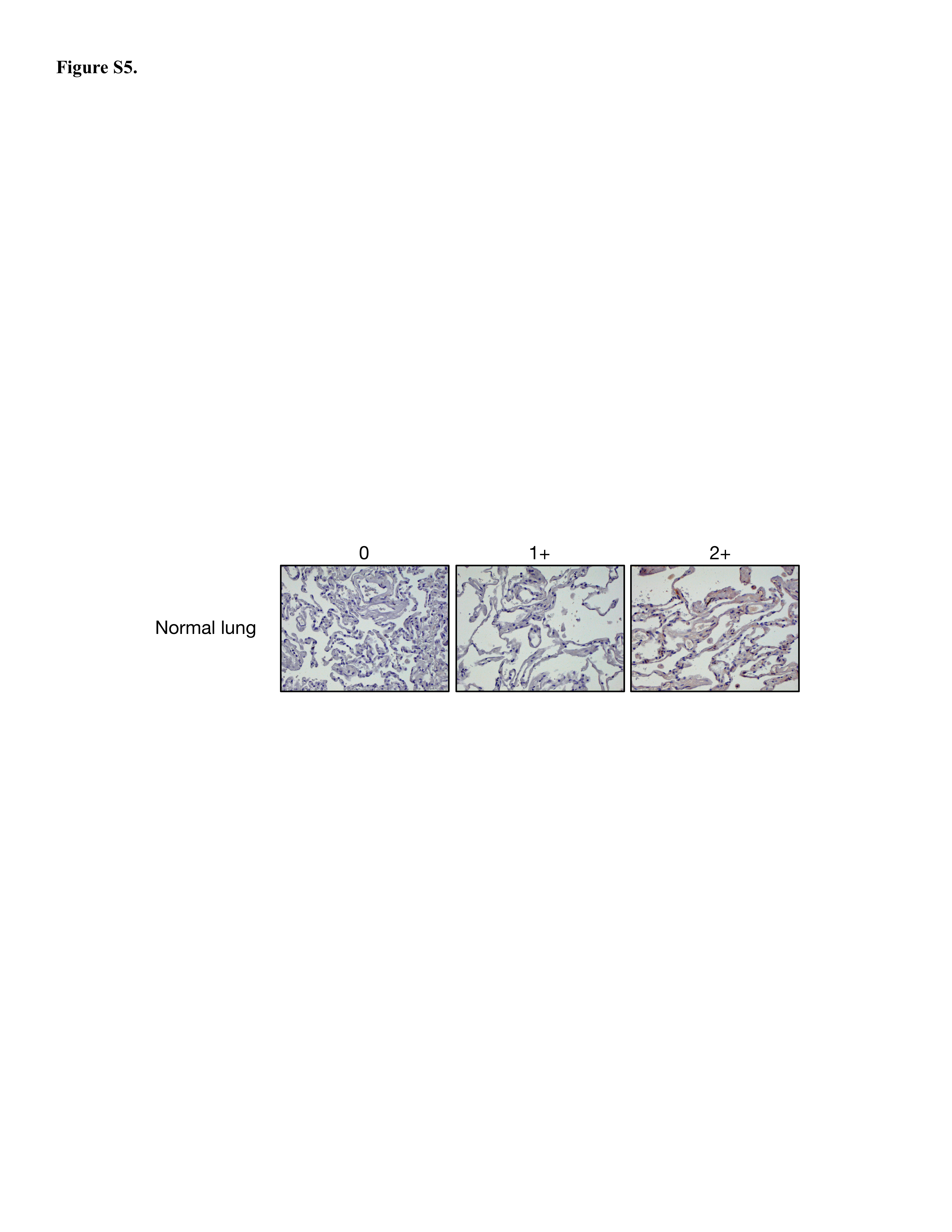

Supplement: Figure S5 — EphB4 protein expression in adjacent normal human lung cancer tissues. Representative immunohistochemistry images of EphB4 expression in normal tissue adjacent to tumor foci. Pathological scoring is indicated above images. (TIFF) [file pone.0067668.s005.tiff]

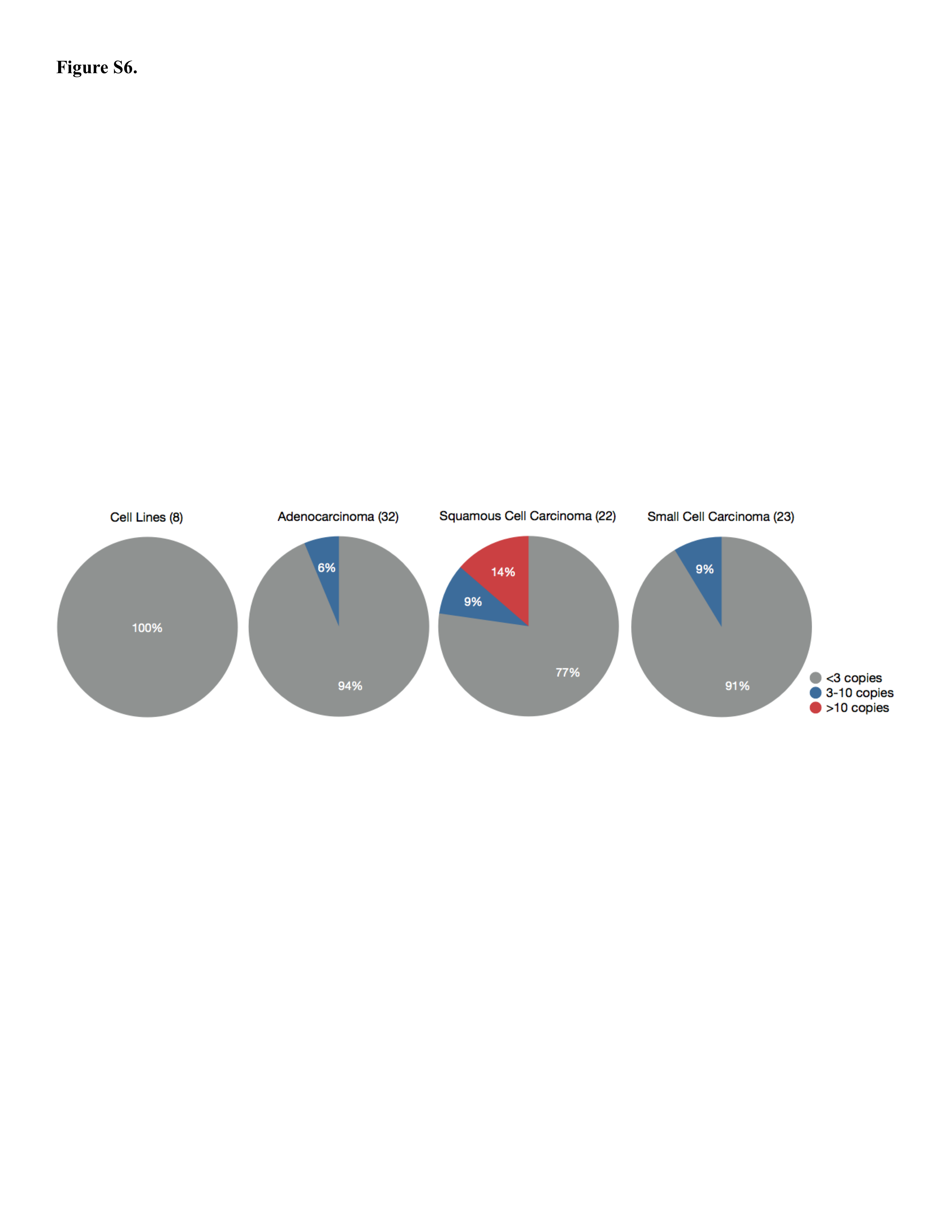

Supplement: Figure S6 — EPHB4 gene copy numbers in human lung cancer tissues and cell lines. The number of tested samples within each subtype or source is denoted in parentheses. (TIFF) [file pone.0067668.s006.tiff]

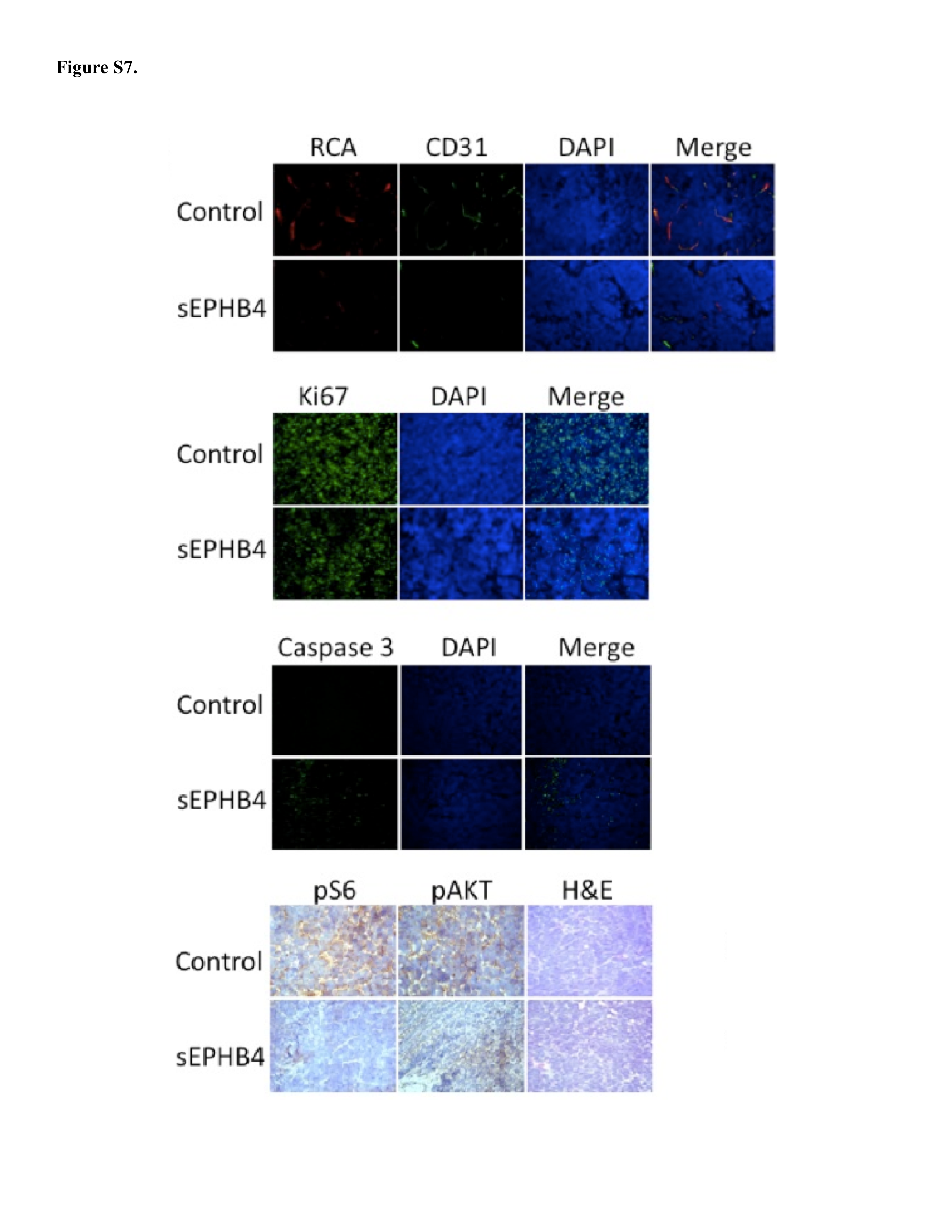

Supplement: Figure S7 — Expression of tumor-associated biomarkers in SCLC tumor xenografts treated with soluble EphB4 in vivo . RCA-lectin, CD31, Ki-67, and caspase-3 staining are demonstrated by immunofluorescence. DAPI was used as a nuclear counterstain. Phosphorylated S6 and phosphorylated Akt expression are demonstrated by immunohistochemistry. (TIFF) [file pone.0067668.s007.tiff]

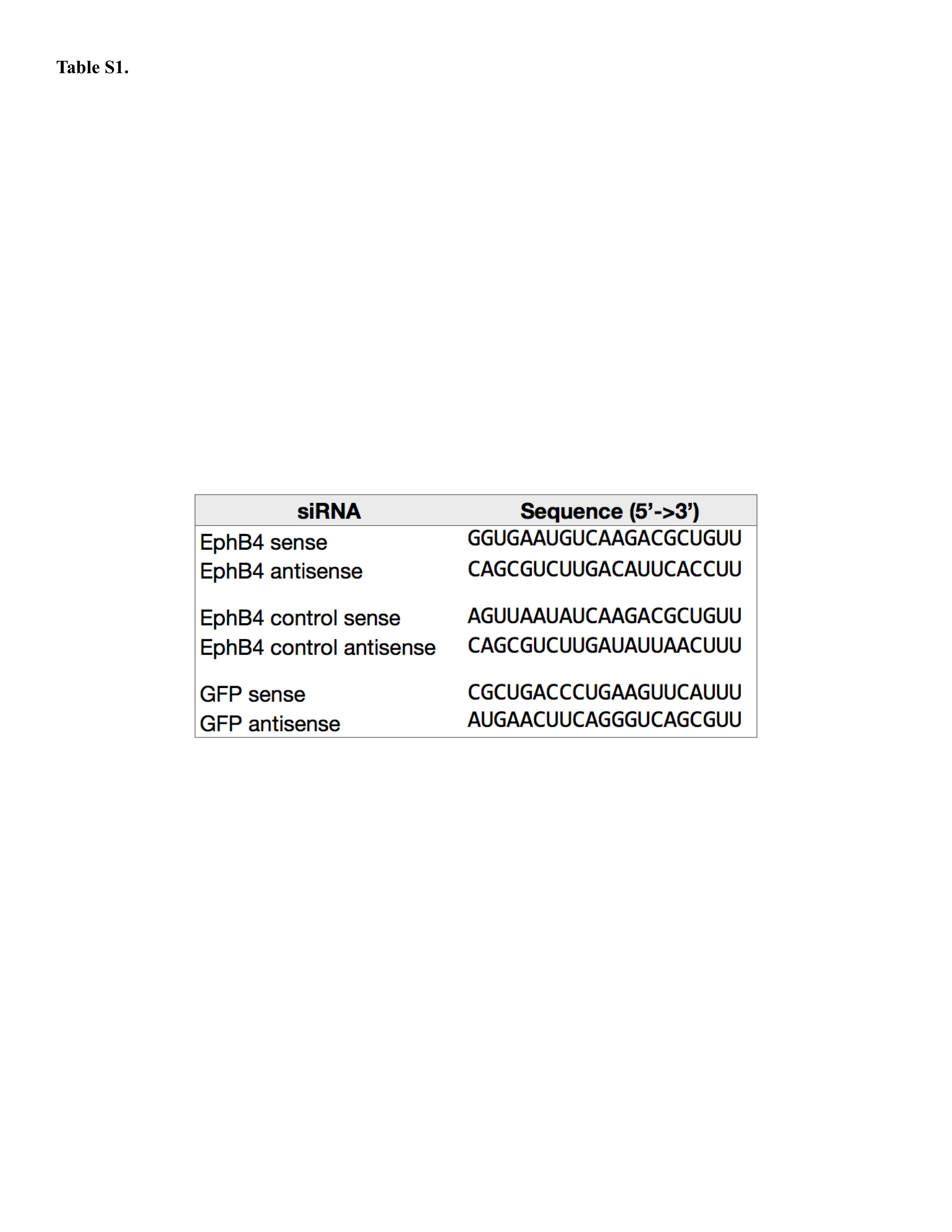

Supplement: Table S1 — Sequences of siRNA oligonucleotides used for gene knockdown. (TIFF) [file pone.0067668.s008.tiff]
